# Hip-Flexor-Suppressing Pendular Gait Strategy Facilitates Walking Recovery After Lesser Trochanter Fracture in an Elderly Patient: A Case Report

**DOI:** 10.7759/cureus.110844

**Published:** 2026-06-14

**Authors:** Shun Shiromoto, Masaaki Nakajima

**Affiliations:** 1 Rehabilitation, Hakuai Medical Corporation Kisaka Hospital, Higashi-Hiroshima City, JPN; 2 Physical Therapy, School of Health Science and Social Welfare, Kibi International University, Takahashi, JPN

**Keywords:** elderly, gait strategy, hip flexor, hip fracture, japanese geriatrics, lesser trochanter fracture, pelvic rotation, rehabilitation

## Abstract

Delayed recovery of limb advancement is a common challenge after lesser trochanter fractures due to impaired hip flexor function. We report a case of an elderly patient who regained walking ability using a hip-flexor-suppressing pendular gait strategy.

A 79-year-old man sustained a femoral neck fracture with concomitant lesser trochanter avulsion. Postoperatively, active hip flexion was limited, and conventional gait patterns were difficult. Instead, the patient adopted a compensatory strategy characterized by trunk forward inclination and pelvic rotation, enabling pendular advancement of the affected limb without active hip flexion.

Gait observation revealed a reduced step length and a prolonged stance phase on the affected limb, suggesting its role as a stable pivot for limb advancement. This strategy minimized pain and mechanical stress at the iliopsoas insertion while facilitating functional ambulation.

This case highlights that limb advancement during gait does not necessarily depend on active hip flexion. Rehabilitation strategies focusing on movement adaptation rather than muscle strengthening alone may be effective in elderly patients with lesser trochanter fractures.

## Introduction

Lesser trochanter fractures can impair hip flexor function due to the insertion of the iliopsoas muscle, which plays a critical role in limb advancement during gait [1]. In elderly patients, hip fractures frequently lead to functional decline, particularly affecting walking ability and independence. Restoration of gait is therefore a primary goal of rehabilitation.

Limb advancement during gait is typically achieved through coordinated hip flexor activation [2]. However, previous biomechanical studies have demonstrated that alternative mechanisms, such as trunk motion and pelvic rotation, can contribute to forward progression of the limb [3,4]. However, clinical reports describing the practical use of such compensatory gait strategies in patients with lesser trochanter fractures remain limited.

This report describes a case in which walking ability was restored using a hip-flexor-suppressing pendular gait strategy, highlighting an alternative approach to gait rehabilitation in the early postoperative phase. In the present report, a pendular gait strategy refers to limb advancement achieved through trunk and pelvic motion rather than direct hip flexor activation.

## Case presentation

**Figure 1:**
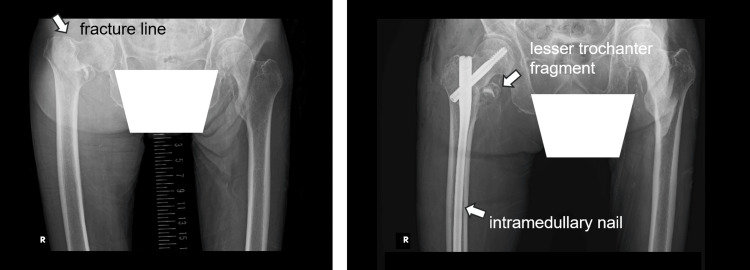
Radiographic findings. (A) Preoperative radiograph showing the right hip fracture. (B) Postoperative radiograph demonstrating intramedullary nail fixation and a lesser trochanter fragment.

A 79-year-old man with a history of chronic heart failure sustained a right hip fracture following a fall and underwent intramedullary nail fixation. Before the injury, he lived independently during the daytime, although family members residing on the same property provided assistance with meals and some daily activities. For indoor mobility, he routinely used a forearm support walker. Two weeks after surgery, he was transferred to our hospital. Postoperative radiographs revealed a fracture of the lesser trochanter (Figure 1A, 1B).

Clinical assessment included pain intensity using an 11-point Numerical Rating Scale (NRS; 0=no pain, 10=worst imaginable pain), clinical gait observation, and walking performance. Walking performance at postoperative week 7 was quantified using a 10-m walk test performed with the forearm-support walker at the patient's comfortable walking speed.

At admission (postoperative week 2), bed mobility, transfers, and wheelchair mobility required full assistance because active hip flexion was severely limited. Pain intensity was assessed using an 11-point NRS during movement. Groin pain was provoked by hip flexion and was rated as 7 on the NRS. Advancement of the affected limb during gait was difficult. Clinical gait observation performed by experienced physical therapists revealed trunk forward inclination, reduced step length, prolonged stance phase on the affected limb, mild knee flexion during stance, and decreased walking speed. Observation was performed visually during overground walking with the assistance of the therapist.

Because the iliopsoas inserts at the lesser trochanter, active hip flexion was intentionally minimized to reduce traction stress at the fracture site. Limb advancement during gait was facilitated using trunk and pelvic movement rather than active hip flexion [4].

The intervention included quadriceps isometric exercises, pelvic rotation exercises, and gait training designed to facilitate trunk forward inclination. During gait training in the parallel bars, the bar height was adjusted slightly lower than usual, and the patient was verbally instructed to lean the trunk forward. Subsequently, gait training was performed using a forearm-support walker adjusted to a relatively low height to encourage maintenance of the forward-inclined trunk posture. Straight leg raise and active hip flexion exercises were avoided.

Physical therapy was performed five days per week for approximately 40 minutes per session. All rehabilitation interventions were provided and supervised by licensed physical therapists.

At postoperative week 3, gait training was initiated in parallel bars using a step-to gait pattern. Advancement of the affected limb remained difficult, and pain was rated as NRS 5. At postoperative week 4, the patient became able to advance the affected limb several centimeters during gait, accompanied by a reduction in pain to NRS 4. At postoperative week 5, pendular gait training using a forearm-support walker was introduced. The patient was able to walk approximately 10 m within the rehabilitation room. At postoperative week 6, walking distance increased to 25 m and pain improved to NRS 3. The patient became able to advance the affected limb beyond the contralateral limb during gait.

Walking performance was quantified using a 10-m walk test performed with the forearm-support walker at the patient's comfortable walking speed. At postoperative week 7, gait speed further improved, and the patient completed the 10-m walk test in 43.6 seconds (0.23 m/s) using the forearm-support walker. Pain decreased to NRS 2. The patient's clinical course and functional recovery are summarized in Table 1.

**Table 1:** Clinical Timeline NRS: Numerical Rating Scale.

Postoperative week	Clinical status
Week 2 (transfer)	Total assistance for bed mobility and transfers. Active hip flexion severely limited. NRS 7.
Week 3	Parallel-bar gait training initiated. Advancement of the affected limb difficult. NRS 5.
Week 4	Affected-limb advancement possible for several centimeters. NRS 4.
Week 5	Pendular gait training with forearm-support walker initiated. Walking 10 m achieved.
Week 6	Walking distance increased to 25 m. NRS 3. Affected limb could be advanced beyond the contralateral limb.
Week 7	Walking speed improved. 10-m walking test: 43.6 s. NRS 2.

## Discussion

This case demonstrates that walking ability can be restored through a hip-flexor-suppressing pendular gait strategy after a lesser trochanter fracture. In such fractures, hip flexion is often impaired due to pain and traction stress at the iliopsoas insertion [1,2]. Limb advancement during gait is typically achieved through hip flexor activation [3]; however, it can also be achieved through trunk movement and pelvic rotation [4]. This case suggests that limb advancement does not necessarily depend on active hip flexion.

While natural recovery cannot be excluded, the temporal relationship between the intervention and improvement suggests a contribution of the therapeutic strategy. Clinically, pain improved from NRS 7 at admission to NRS 2 at postoperative week 7, while walking ability progressed from complete dependence for mobility to 10-m ambulation using a forearm-support walker at a walking speed of 0.23 m/s.

**Figure 2:**
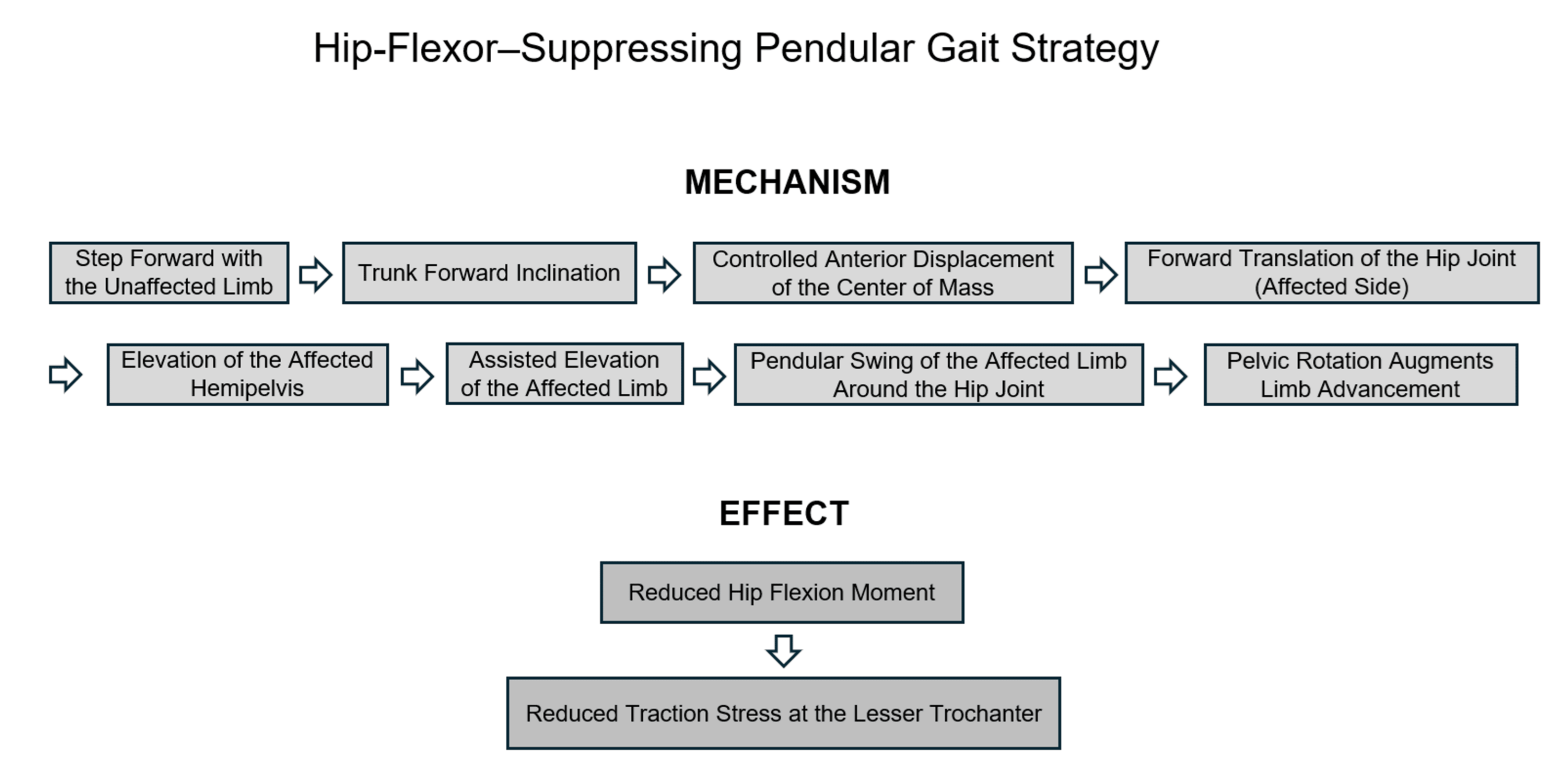
Hip-Flexor–Suppressing Pendular Gait Strategy Conceptual model of a hip-flexor-suppressing gait strategy. Following a step forward with the unaffected limb to establish a stable base of support, trunk inclination induces controlled anterior displacement of the center of mass. This leads to forward translation of the hip joint on the affected side and elevation of the affected hemipelvis, enabling assisted elevation and pendular swing of the limb. Pelvic rotation further augments limb advancement. This mechanism reduces the hip flexion moment and consequently reduces traction stress at the lesser trochanter. Image created by the authors.

**Figure 3:**
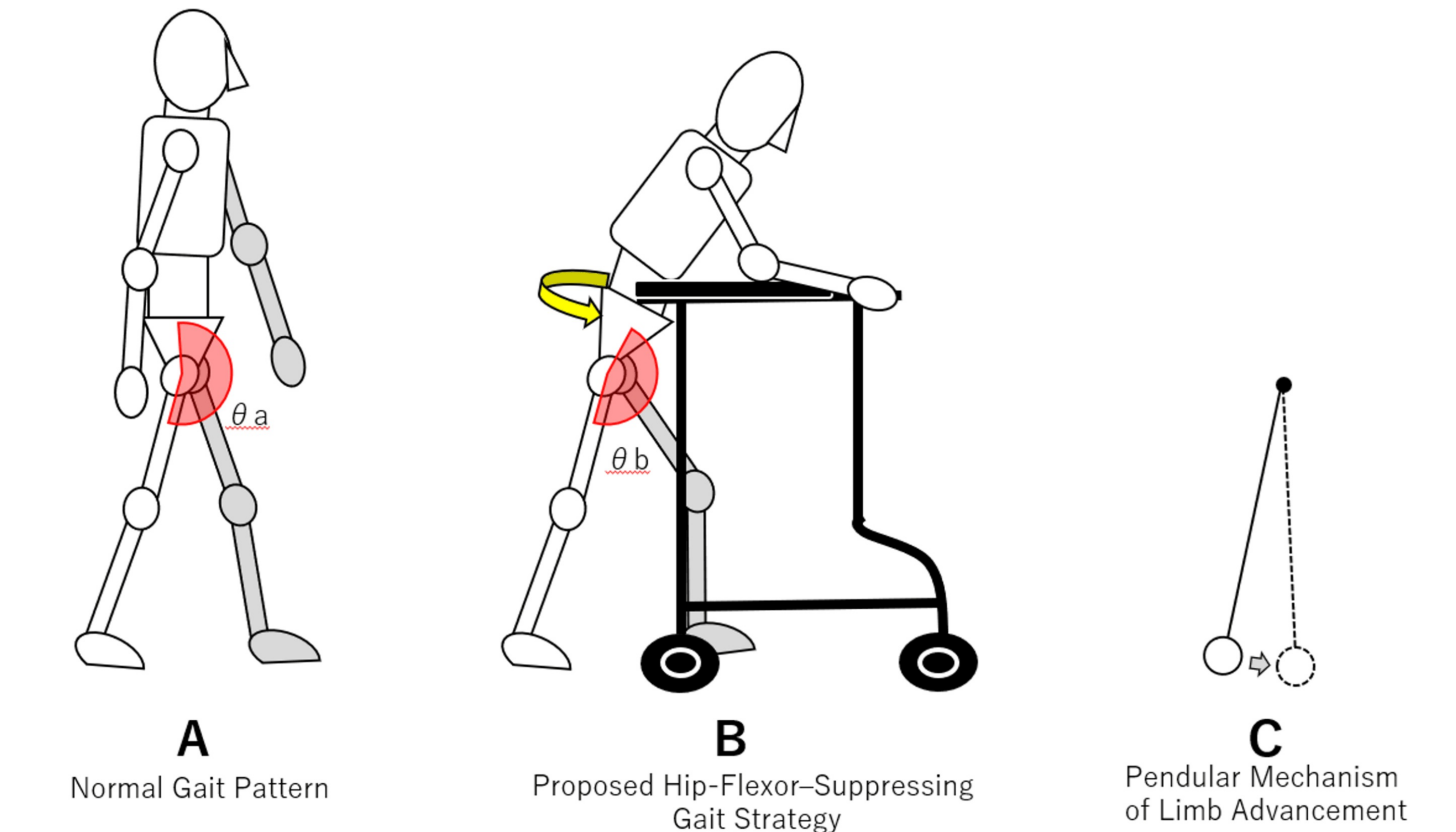
Proposed hip-flexor-suppressing pendular gait strategy following lesser trochanter fracture. (A) In a normal gait pattern, forward movement of the trunk after advancement of the unaffected limb places the affected hip in relative extension (θa), which may increase traction stress at the lesser trochanter through tension generated by the iliopsoas. (B) In the proposed gait strategy, use of a forearm support walker with trunk forward inclination helps maintain the affected hip in relative flexion during gait (θb). After advancement of the unaffected limb and controlled anterior displacement of the center of mass, elevation and anterior rotation of the affected hemipelvis facilitate pendular advancement of the affected limb while minimizing active hip flexion. This strategy may reduce traction stress at the lesser trochanter. (C) Conceptual illustration of the pendular mechanism of limb advancement. The affected hip joint functions as the pivot point, while the affected lower limb acts as the pendular segment. Limb advancement is achieved through pendular motion and pelvic movement rather than direct hip flexor activation. Image credit: Created by the authors using Microsoft PowerPoint (Microsoft, Redmond, WA).

The gait characteristics observed in this case can be interpreted as a coordinated movement strategy rather than isolated compensations. Trunk forward inclination shifted the center of mass anteriorly, reducing the need for active hip flexion. Mechanistically, anterior shift of the center of mass reduces the hip flexion moment required for limb advancement. This biomechanical adjustment allows limb progression through passive pendular motion rather than active muscle-driven flexion and redistributes the workload from hip flexion to trunk-pelvis dynamics. This facilitated pelvic rotation, which enabled pendular advancement of the swing limb. In this context, limb advancement was achieved through trunk-pelvis coupling rather than hip flexor activation (Figures 2, 3).

Importantly, this movement strategy likely reduces traction stress at the lesser trochanter by minimizing direct activation of the iliopsoas. This reduction in mechanical stress at the fracture-related insertion site may explain the observed pain relief and functional improvement, as reflected by the progressive reduction in NRS scores from 7 at admission to 2 at postoperative week 7.

The prolonged stance phase on the affected limb suggests that the limb functioned as a stable pivot for pelvic rotation rather than as a propulsive limb, indicating a shift from a hip-flexion-driven gait to a stance-driven gait strategy. The reduced step length can be interpreted as a strategy to limit limb excursion and further reduce traction stress at the lesser trochanter. Mild knee flexion during stance may have contributed to shock absorption and facilitated smoother pendular motion.

Figure 3 visually summarizes the proposed pendular gait strategy. Trunk forward inclination, maintenance of relative hip flexion, and pelvic rotation may collectively facilitate limb advancement while reducing iliopsoas traction stress.

Taken together, these findings indicate that the patient adopted a hip-flexor-suppressing pendular gait strategy in which trunk inclination, pelvic rotation, and stance stability were integrated to achieve safe limb advancement while reducing mechanical stress at the lesser trochanter.

The compensatory gait pattern observed in this case was likely a temporary adaptation during the healing phase rather than a permanent gait strategy. As pain gradually decreased and walking ability improved, the patient became able to advance the affected limb beyond the contralateral limb and increased walking speed. These findings suggest that the pendular gait strategy served as a transitional mechanism that enabled functional ambulation while minimizing traction stress at the lesser trochanter during fracture healing.

The long-term consequences of maintaining this compensatory gait pattern remain unknown because follow-up beyond the early postoperative period was not performed. Potential adverse effects, including increased lumbar loading and secondary musculoskeletal symptoms, should be investigated in future studies.

It should be noted that the patient used a forearm support walker before the injury. Therefore, the present findings should be interpreted within the context of an individual who already relied on assisted ambulation prior to fracture.

This case is limited by its single-subject design and the lack of detailed objective gait analysis, including kinematic measurements and comprehensive spatiotemporal gait assessment. Future studies incorporating quantitative gait assessment are needed to validate the proposed mechanism.

## Conclusions

This case demonstrates that walking ability can be restored through a hip-flexor-suppressing pendular gait strategy following a lesser trochanter fracture. Rather than relying on active hip flexion, limb advancement was achieved through trunk forward inclination and pelvic rotation, enabling safe and pain-free gait while reducing traction stress at the lesser trochanter.

These findings suggest that rehabilitation strategies focusing on movement adaptation may be effective in elderly patients when conventional muscle-driven approaches are limited. This strategy may offer a practical alternative for managing fracture-related stress at the lesser trochanter in the early postoperative phase. Future studies are needed to validate this strategy and to determine its applicability to broader patient populations.
